# miR-202 Suppresses Cell Proliferation by Targeting FOXR2 in Endometrial Adenocarcinoma

**DOI:** 10.1155/2017/2827435

**Published:** 2017-07-30

**Authors:** Xinchao Deng, Congzhe Hou, Zhen Liang, Huali Wang, Lin Zhu, Hui Xu

**Affiliations:** Department of Obstetrics and Gynecology, The 2nd Hospital of Shandong University, Jinan, Shandong 250012, China

## Abstract

**Background:**

MicroRNA-202 (miR-202) has been reported to be aberrantly regulated in several cancers. The aim of this study is to explore the functional role of miR-202 in EAC tumor growth.

**Material and Methods:**

miR-202 expression was detected by qRT-PCR. TargetScan and luciferase reporter assay were used to elucidate the candidate target gene of miR-202. The FOXR2 protein level was assessed by Western blot and immunohistochemistry. Survival analysis was explored for FOXR2 expression in EAC patients.

**Results:**

miR-202 expression was significantly decreased in EAC tissues (*P* < 0.01) compared with that in control tissues. And the downregulate miR-202 was significantly associated with poor prognosis (*P* < 0.01). Re-expression of miR-202 dramatically suppressed cell proliferation in vitro and tumor growth in vivo. FOXR2 was identified as a direct target of miR-202. In EAC tissues, FOXR2 was upregulated and the increased FOXR2 was significantly associated with poor prognosis. In miR-202-transfected cells, the FOXR2 expression was inversely changed. The analysis of FOXR2 protein expression and miR-202 transcription in EAC tissues showed negative correlation (*R* = −0.429).

**Conclusion:**

miR-202 may function as a tumor suppressor in EAC tumor growth by targeting FOXR2 oncogene, which may provide new insights into the molecular mechanism and new targets for treatment of EAC.

## 1. Background

Endometrial cancer (EC) is the most common malignancy of the female genital tract [1]. Generally, EC is classified as type I endometrioid EC (accounts for 70 ~ 80% of new diagnosed cases) and type II nonendometrioid EC based on etiology and clinical variables. Endometrial adenocarcinoma (EAC) is identified as type I endometrioid EC, which are associated with good prognosis. However, cell proliferation and migration decrease the survival rates of patients after surgical treatment. Thus, to further improve patient's survival rate, it is clinically urgent to understand the molecular mechanism of EAC development.

MicroRNAs (miRNAs) are endogenous noncoding small RNAs of 19–22 nucleotides that function as gene regulators at posttranscriptional level by directly binding to the 3′untranslated regions (3′-UTR) of messenger RNA (mRNA), resulting in the degradation of mRNA or inhibition of transcription. To date, increasing evidences indicated that miRNAs perform important roles in the development of cancers, such as tumor cell proliferation, invasion, or apoptosis, functioning as tumor suppressors or oncogenes [2]. Many studies have proposed that miR-202 is a tumor suppressor as its expression has been found downregulated in many kinds of cancers. In esophageal squamous cell carcinoma, miR-202 inhibits tumor progression by targeting LAMA1 [3]. In colon cancer, miR-202 was downregulated and was associated with colon cancer pathways and correlated with cyto- or chemokine expression [4]. In the serum of multiple myeloma patients, the relative expression of miR-202 was significantly higher than that in healthy controls [5]. Except for the role as a tumor suppressor in tumor, it has been identified that in multiple myeloma, miR-202 may correlate with drug sensitivity [6]. In gastric cancer, miR-202 was demonstrated inhibiting cell proliferation and apoptosis [7]. In human colorectal carcinoma, microRNA-202-3p inhibits cell proliferation by targeting ADP-ribosylation factor-like 5A [8]. In osteosarcoma, miR-202 acts as a novel tumor suppressor to regulate cell proliferation and apoptosis by downregulating Gli2 expression [9]. Besides, miR-202 was found suppressing cell proliferation in human hepatocellular carcinoma [10], nonsmall cell lung cancer [11], and cervical cancer [12]. But the miR-202 is rarely understood in the development and progression of EAC.

human forkhead box (FOX) proteins are a family of transcription factors, containing at least 43 members, which are implicated in a wide range of biological processes, including migration, apoptosis, differentiation, proliferation, and metabolism [13, 14]. The FOX proteins are related through the presence of an evolutionary conserved “forkhead” or “winged-helix” DNA-binding domain (DBD) [13]. FOXR2, also known as FOXN6, was firstly identified at RP11-167p23, containing a highly conserved forkhead domain at its C-terminus [15]. Recent studies proposed that FOXR2 was aberrantly expressed in several cancers. In breast cancer, Katoh et al. showed that FOXR2 was upregulated and high expression of FOXR2 was significantly correlated with poor prognosis [15]. In prostate cancer, FOXR2 was overexpressed in prostate cancer cell lines and the knockdown of FOXR2 significantly repressed the proliferation, migration, and invasiveness of prostate cancer cells [16]. Wang et al. showed that FOXR2 promoted cell growth and colony formation, whereas knockdown of FOXR2 by RNA inference inhibited cell growth and decreased the growth ability of HCC cells [17]. However, the expression and function of FOXR2 in endometrial cancer are rarely understood.

In this study, we provide evidences that miR-202 was aberrantly downregulated in EAC tissues compared with that in nontumor tissues. Re-expression of miR-202 significantly suppressed cell proliferation both in vitro and vivo. FOXR2 was identified as a direct and functional target of miR-202. These findings may provide potential insights into the molecular mechanism and new targets for EAC treatment.

## 2. Material and Methods

### 2.1. Tissue Samples

A total of 90 endometrioid adenocarcinoma tissues and 40 corresponding nontumor tissues were collected at the Department of Obstetrics and Gynecology, The 2nd Hospital of Shandong University, from January 2008 to June 2012. The nontumor tissues were normal endometrium from patients who underwent hysterectomy because of benign diseases, such as leiomyoma. Tissues obtained from patients at the time of surgical resection were immediately stored at −80°C. None of the patients have received the radiotherapy or chemotherapy before surgery. The experiment was approved by the Ethics Committee of The 2nd Hospital of Shandong University.

### 2.2. Cell Lines

Two human endometrioid adenocarcinoma cell lines (KLE and AN3CA) were cultured in Dulbecco's Modified Eagle's Medium (HyClone, South Logan, UT, USA) supplemented with 10% fetal bovine serum (HyClone) and 100 U/mL penicillin/streptomycin. All cells were maintained in a humidified chamber with 5% CO_2_ at 37°C.

### 2.3. Quantitative Real-Time PCR

Total RNA was extracted using TRIzol Reagents (Invitrogen, Carlsbad, CA, USA) according to the manufacturer's instruction. Quantitative real-time PCR (qRT-PCR) was performed to detect the relative expression of miR-202 in cells and tissues using the NCode miRNA qRT-PCR Analysis (Invitrogen, CA) according to the manufacturer's protocols. The snRNA U6 was used as internal control.

### 2.4. Western Blot Assay

Total protein was extracted from cells or tissues using M-PER mammalian protein extraction reagent (Pierce Biotechnology, MA). Protein concentration was measured using BCA Protein Assay kit (Beyotime). The proteins were separated by 10% SDS-PAGE and transformed to nitrocellulose filter membranes (Hybond, Escondido, CA, USA). After blocked with 5% skimmed milk for 1 h at room temperature, the membrane was incubated with the first antibody at 4°C overnight, followed by the incubation with the second antibody for 1 h at room temperature. The protein bonds were visualized using EZ-ECL chemiluminescence detection kit for HRP (Biological Industries, Beit-Haemek, Israel). All the antibodies were purchased from Abcam.

### 2.5. Cell Transfection

miR-202 inhibitor and the negative control were transfected into cells using the lipofectamine RNAi MAX (Invitrogen, Carlsbad, CA, USA) following the manufacturer's protocols at a final concentration of 50 nM. Cells were harvested after 48 h of transfection for further analysis. Vectors expressing GIPZ-miR-202 or control vectors were packaged to produce lentivirus. Virus infection and puromycin selection was conducted to get stable cell lines.

### 2.6. Luciferase Reporter Assay

For luciferase reporter assay, FOXR2-3′-UTR sequences were amplified and cloned into the downstream of the luciferase reporter gene in pMIR-REPORT luciferase vectors. Mutant 3′-UTR of FOXR2 [18] was generated using QuikChange site-directed mutagenesis kit (Stratagene, Cedar Creek, TX, USA). Cells were collected after 48 h of transfection with miR-202 mimics or control plus pMIR-FOXR2-3′-UTR (WT) or pMIR-FOXR2-3′-UTR [18]. Luciferase activity was measured by a dual-luciferase reporter assay system (Promega, GenePharma, Shanghai). Firefly luciferase activity was normalized to Renilla luciferase activity. All experiments were performed in triplicate.

### 2.7. Immunohistochemistry Assay

The 90 EAC tissues and 40 control tissues underwent fixation in 4% paraformaldehyde, paraffin embedded, and cut into 5 *μ*m sections. Tissue slides were deparaffinized in xylene and rehydrated in graded series of ethanol. After antigen retrieval was performed to the sections, they were blocked by 3% hydrogen peroxidase in methanol. The slides were then blocked with goat serum and incubated with FOXR2 primary antibody. Secondary antibody was added and incubated at room temperature. After Streptavidin HRP was added, sections were stained with DAB substrate and counterstained with hematoxylin. Staining intensity was graded and each section was scored as 0 to 3. The tissues with a score of 0 and 1 were defined as FOXR2 negative while tissues with a score of 2 and 3 were defined as negative.

### 2.8. Cell Proliferation Assay

Cell proliferation was measured using 3-(4, 5-dimethylthiazol-2-yl)-2, 5-diphenyltetrazolium bromide (AMRESCO, Solon, OH, USA) assay. Cells transfected with GIPZ-miR-202, miR-202 inhibitor, or control were cultured in 96-well plates for 1–7 days. On the indicated days, cells were added with 3-(4, 5-dimethylthiazol-2-yl)-2, 5-diphenyltetrazolium bromide and incubated for 4 h at 37°C. Then, the supernatants were removed and DMSO (150 *μ*L/well) was added into wells to dissolve formazan crystals. Absorbance was detected at 490 nm for each sample using a Multilabel Plate Reader (PerkinElmer, Waltham, MA, USA).

### 2.9. Tumor Growth Assay In Vivo

Four-week-old female BALB/C athymic nude mice were used in the study for tumor growth assay in vivo. A total of 5 × 10^6^ KLE cells transfected with GIPZ-miR-202 or negative control were injected into flanks of mice (*n* = 5 in each group). Tumor size was measured once a week after 14 days injection. Mice were sacrificed 49 days after injection. Tumors were excised and measured. The tumor size was calculated according to the standard formula: tumor volumes (cm^3^) = (the longest diameter) × (the shortest diameter)^2^ × 0.5. All the experiments were approved by the Ethics Committee of The 2nd Hospital of Shandong University.

### 2.10. Statistical Analysis

Statistical analyses were performed using SPSS 13.0 software package. All data were presented as mean ± SD. The groups were compared using Student *t*-test unless otherwise noted. Patient's survival rate was measured using Kaplan-Meier analyses. Chi-square (v2) and Fisher's exact test were used to measure the relationship between the miR-202 and FOXR2 expression. Difference was considered significant at *P* < 0.05.

## 3. Results

### 3.1. miR-202 Is Frequently Downregulated in EAC Tissues and Is Associated with Overall Survival Rate

**Figure 1:**
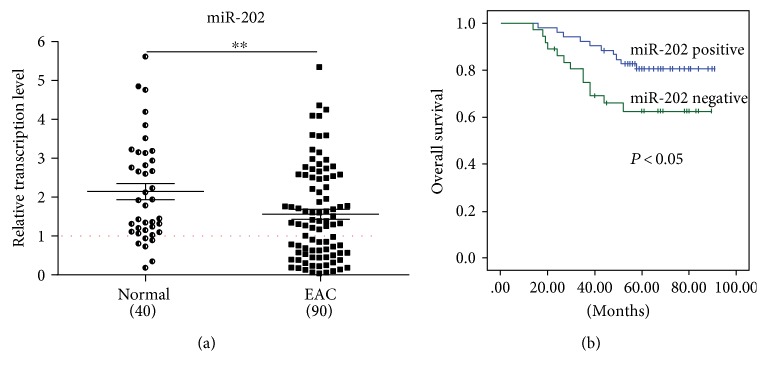
miR-202 is downregulated in EAC tissues and decreased miR-202 is significantly associated with poor prognosis. (a) Compared with that in control tissues (*n* = 40), miR-202 was frequently downregulated in EAC tissues (*n* = 90). (b) Low expression of miR-202 was significantly associated with poor prognosis. ^∗∗^*P* < 0.01.

To investigate the relative expression of miR-202 in EAC tissue, we performed qRT-PCR in 90 EAC tissues and 40 control tissues. The results showed that compared with control tissues, miR-202 was significantly downregulated in EAC tissues (*P* < 0.01) (Figure 1(a)), indicating that miR-202 may be a putative tumor suppressor.

Besides, to understand the prognostic significance of downregulated miR-202 in EAC patients, we assessed the overall survival rate in these 90 patients. 90 patients were divided into two groups according to the expression level of miR-202. The mean value of miR-202 expression in the EAC group was defined as the cutoff (38 EAC cases were positive and 52 EAC cases were negative). Significant difference was found in patient overall survival rate between the miR-202 positive group and negative group (*P* < 0.05) (Figure 1(b)), indicating that downregulated miR-202 correlated with poor prognosis.

### 3.2. Re-Expression of miR-202 Dramatically Decreases EAC Growth In Vitro and In Vivo

**Figure 2:**
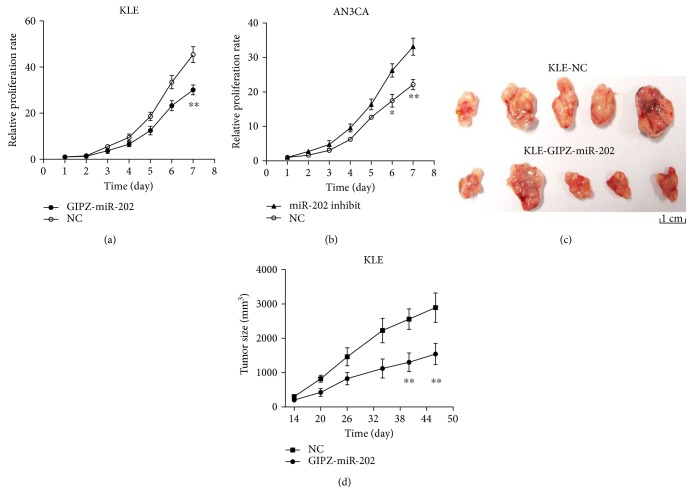
Re-expression of miR-202 significantly inhibited tumor growth. (a) Re-expression of miR-202 in KLE cells significantly suppressed cell proliferation. (b) miR-202 inhibitor significantly promoted AN3CA cell proliferation. (c, d) Re-expression of miR-202 greatly suppressed tumor growth in vivo. ^∗^*P* < 0.05, ^∗∗^*P* < 0.01.

To further understand the biological function of miR-202 in the proliferation of EAC, KLE cells transfected with GIPZ-miR-202 and AN3CA cells transfected with miR-202 inhibitor were used to assay the effect of miR-202 on the cell proliferation. The successful re-expression or knockdown of miR-202 was determined by qRT-PCR. The MTT assay showed that re-expression of miR-202 significantly suppressed cell proliferation in KLE cells (Figure 2(a)) and knockdown of miR-202 significantly promoted cell proliferation in AN3CA cells (Figure 2(b)).

Then, the potential tumor-inhibitory effect of miR-202 was assessed in vivo. KLE cells stably expressed with GIPZ-miR-202 were injected into the flanks of mice. And tumor size was measured weekly. Compared with the NC group, re-expression of miR-202 significantly decreased tumor growth (Figure 2(d)). After 49 days inoculation, tumors were obtained for comparison between the NC group and miR-202 group (Figure 2(c)). The tumors were much smaller in the miR-202 group.

These findings indicated that miR-202 significantly suppressed EAC tumor growth both in vitro and vivo.

### 3.3. miR-202 Downregulates FOXR2 by Directly Targeting Its 3′-UTR

**Figure 3:**
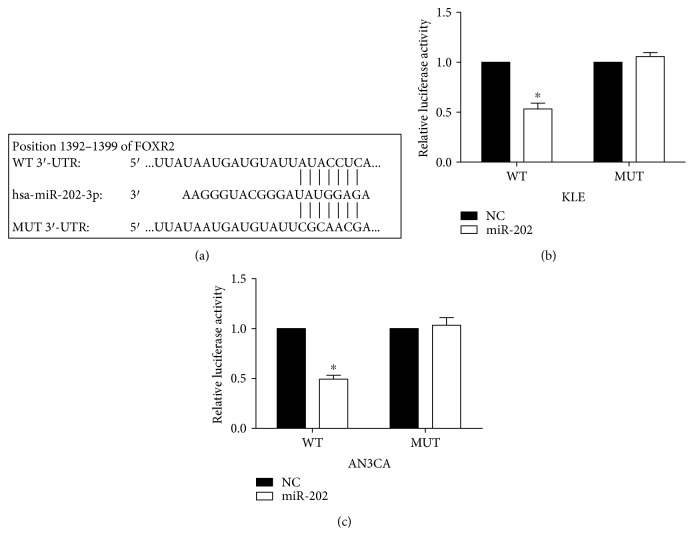
FOXR2 is a direct target of miR-202. (a) The putative binding site of miR-202 in the 3′-UTR of FOXR2. The mutation was generated using QuikChange site-directed mutagenesis kit. (b, c) Analysis of luciferase activity. The relative luciferase activity was reduced when cells were cotransfected with miR-202 mimic and pMIR-FOXR2-3′-UTR (WT), and it was rescued when cells were cotransfected with miR-202 mimic and pMIR-FOXR2-3′-UTR [18]. Firefly luciferase activity was normalized to Renilla luciferase activity. ^∗^*P* < 0.05.

**Figure 4:**
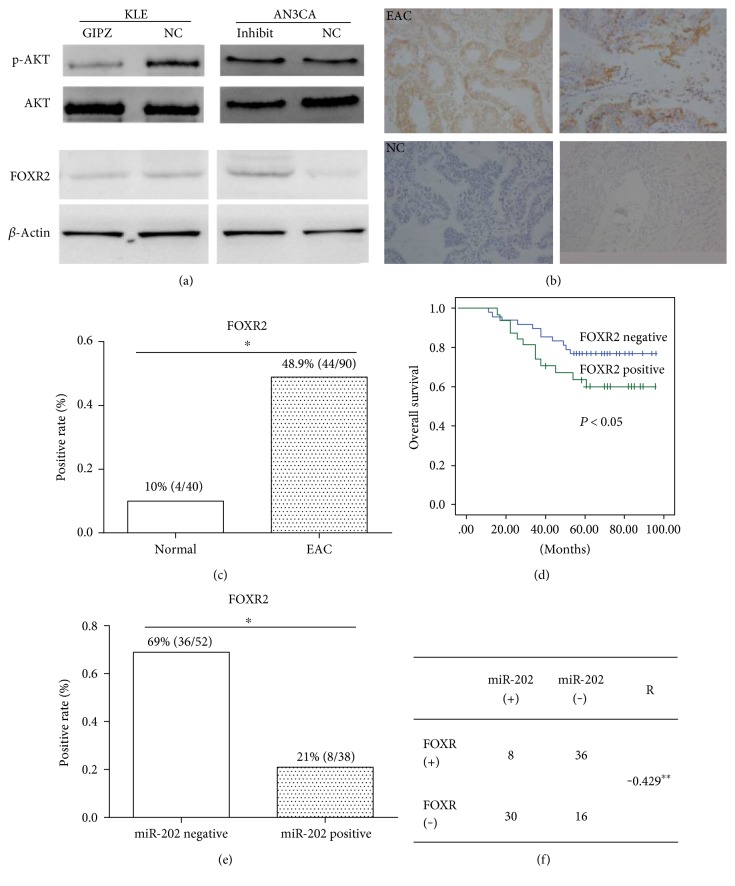
FOXR2 is upregulated in EAC and is associated with poor prognosis. (a) Expression of FOXR2 and p-AKT was downregulated by miR-202 in KLE cells and was upregulated by miR-202 inhibitor in AN3CA cells assayed by Western blotting. *β*-Actin was used as internal control. (b, c) Positive expression of FOXR2 in EAC is higher compared with that in control tissues. (d) High FOXR2 expression level was significantly associated with poor prognosis. (e) In miR-202-positive EAC tissues, the FOXR2-positive rate is significantly lower than that in miR-202-negative tissues. (f) Data illustrates the FOXR2-positive cases in miR-202-positive and miR-202-negative EAC tissues. ^∗^*P* < 0.05, ^∗∗^*P* < 0.01.

To investigate the downstream of miR-202, we searched for the TargetScan website. FOXR2 was found as a putative target of miR-202 and the potential binding sites were found at 1392–1399 nt of FOXR2. To verify the target, luciferase reporter was performed in both KLE and AN3CA cells. The mutant [18] putative binding site was generated using QuikChange site-directed mutagenesis kit (Figure 3(a)). Luciferase activity reporter assay (Figure 3(b)) showed that relative luciferase activity was significantly decreased the when cells were cotransfected with miR-202 and pMir-FOXR2-3′UTR (WT) compared with cells cotransfected with miR-control and pMir-FOXR2-3′UTR (WT). The relative luciferase activity was rescued when cells were cotransfected with miR-202 and pMir-FOXR2-3′UTR [18]. We further detected the inhibition effect of miR-202 on FOXR2 expression at protein level. Western blot assay showed that re-expression of miR-202 decreased FOXR2 expression and miR-202 inhibitor increased FOXR2 expression (Figure 4(a)). Meanwhile, P-AKT expression was detected in these transfected cells, and we found that the P-AKT expression level was correlated with FOXR2 expression, implying the proliferation ability change of these cells. These findings indicate that FOXR2 is a direct target of miR-202.

### 3.4. FOXR2 Is Upregulated in EAC and Is Associated with Poor Prognosis

Generally, FOXR2 functions as an oncogene. We first detected the relative expression of FOXR2 in EAC tissues (*n* = 90) and nontumor tissues (*n* = 40) by IHC and found that FOXR2 expression was frequently higher in EAC tissues (Figure 4(b)). The FOXR2 expression between the two group was significantly different; it was frequently upregulated in the EAC group (48.9% positive) compared with that in the control group (10% positive) (Figure 4(c)). The overall survival rate was compared between FOXR2-positive and FOXR2-negative groups divided according to IHC result. The Kaplan-Meier analyses showed that upregulated FOXR2 was significantly associated with poor prognosis (Figure 4(d)). In Figures 4(e) and 4(f), the IHC expression of FOXR2 was assayed in both miR-202-positive and miR-202-negative EAC tissues. The data showed that the FOXR2-positive rate was much lower in the miR-202-positive group (21%) when compared with that in the miR-202-negative group (69%) (*P* < 0.05). The FOXR2 protein expression and miR-202 transcription in EAC tissues showed negative correlation (*R* = −0.429, *P* < 0.01). These findings indicate that FOXR2 is upregulated in EAC and is associated with poor prognosis. In addition, the expression of FOXR2 and miR-202 in EAC tissues was inversely correlated, implying that FOXR2 is negatively regulated by miR-202 as target.

## 4. Discussion

Emerging evidences showed that miRNAs participated in the development and progression of cancers. In EAC, many kinds of miRNAs have been found dysregulated. Dysregulation of microRNA-204 mediates migration and invasion of endometrial cancer by regulating FOXC1 [19]. The microRNA-200 family is identified as being upregulated in endometrial carcinoma [20]. Wu et al. demonstrated that upregulation of miR-145 promotes differentiation by repressing OCT4 in human endometrial adenocarcinoma cells [21]. But the function of miR-202 in EC is rarely understood. In this study, we provide evidence that miR-202 was significantly downregulated in EAC tissues compared with that in control samples (*P* < 0.01). And the reduced miR-202 was associated with patient's overall survival rate (*P* < 0.05). In vitro assay showed that re-expression of miR-202 significantly decreased cell proliferation, and knockdown of miR-202 significantly promoted cell proliferation. Besides, re-expression of miR-202 inhibited tumor growth compared with that of the control group. These findings suggest that miR-202 functions as a tumor suppressor in EAC.

FOXR2 is a member of the FOX protein family and plays important roles in the development and progression of cancers. A study of Xu et al. showed that knockdown of FOXR2 suppresses the tumorigenesis, growth, and metastasis of prostate cancer through inhibiting the Wnt/b-catenin signaling pathway [16]. In breast cancer tissues, FOXR2 was detected upregulated and high expression of FOXR2 significantly correlated to poor prognosis as demonstrated by Song et al. [22]. In human medulloblastoma tissues, FOXR2 was overexpressed, and the overexpression of FOXR2 greatly promoted the proliferation of medulloblastoma cells [23]. In line with these studies, we revealed that FOXR2 was upregulated in EAC tissues compared with that in nontumorous samples and was significantly associated with poor prognosis. Besides, we identified FOXR2 as a direct target of miR-202 in EAC cell lines. Re-expression of miR-202-inhibited FOXR2 expression and knockdown of miR-202 increased FOXR2 expression.

AKT has been demonstrated to be associated with cell proliferation, survival, and tumor growth [24]. The activation of Akt is also one of the most frequent alterations observed in multiple human cancer types, including colorectal cancer [25], breast cancer [26], lung cancer [27], and endometrial cancer [28]. Once activated, Akt could influence many factor expression, which are involved in cell cycle progression, by transcription regulation or direct phosphorylation [29], such as FoxO4, which are directly phosphorylated by Akt, thus affecting the cell cycle [30]. Akt also promotes cyclin D1 translation via indirect activation of mTOR, which increases translation of cyclin D1 by activating ribosomal protein S6K and inhibiting eukaryotic translation initiation factor 4E-binding protein (4E-BP), thus increasing eIF4e activity [31]. And in endometrial cancer, the PI3K/Akt pathway is the most frequently altered biochemical pathway as proposed by Pavlidou and Vlahos [32]. Besides, the Ras/PI3K/PTEN/Akt/mTOR cascades were found involved in chemo and hormonal resistance in breast cancer as the constitutively activated Akt-1 gene was more resistant to doxorubicin, etoposide, and 4-OH-tamoxifen [33]. Also, the p53 and Raf/MEK/ERK pathways play key roles in drug sensitivity [34]. In the present study, we provided evidences that re-expression of miR-202 dramatically inhibited p-AKT expression, indicating that miR-202 inhibited tumor growth partially by targeting AKT pathway.

## 5. Conclusions

In conclusion, we demonstrated that miR-202 functions as a tumor suppressor by inhibiting cell growth in EAC via downregulating oncogene-FOXR2 by directly targeting its 3′-UTR. It is suggested that miR-202 may provide new insights into the molecular mechanism of EAC and the new target for the treatment.
